# Supplementary material for: A lysosomal surveillance response to stress extends healthspan
Source: Nat Cell Biol. 2025 Jun 26;27(7):1083–97. doi: 10.1038/s41556-025-01693-y (PMC12270918; doi:10.1038/s41556-025-01693-y)
Supplement: Supplementary file 1 — Reporting Summary [file 41556_2025_1693_MOESM1_ESM.pdf]

nature portfolio

Corresponding author(s): Terytty Yang Li & Johan Auwerx

Last updated by author(s): May 4, 2025

Reporting Summary

Nature Portfolio wishes to improve the reproducibility of the work that we publish. This form provides structure for consistency and transparency in reporting. For further information on Nature Portfolio policies, see our Editorial Policies and the Editorial Policy Checklist.

Statistics

For all statistical analyses, confirm that the following items are present in the figure legend, table legend, main text, or Methods section.

n/a	Confirmed	
☐	☒	The exact sample size (*n*) for each experimental group/condition, given as a discrete number and unit of measurement
☐	☒	A statement on whether measurements were taken from distinct samples or whether the same sample was measured repeatedly
☐	☒	The statistical test(s) used AND whether they are one- or two-sided<br>*Only common tests should be described solely by name; describe more complex techniques in the Methods section.*
☐	☒	A description of all covariates tested
☐	☒	A description of any assumptions or corrections, such as tests of normality and adjustment for multiple comparisons
☐	☒	A full description of the statistical parameters including central tendency (e.g. means) or other basic estimates (e.g. regression coefficient) AND variation (e.g. standard deviation) or associated estimates of uncertainty (e.g. confidence intervals)
☐	☒	For null hypothesis testing, the test statistic (e.g. *F*, *t*, *r*) with confidence intervals, effect sizes, degrees of freedom and *P* value noted<br>*Give P values as exact values whenever suitable.*
☒	☐	For Bayesian analysis, information on the choice of priors and Markov chain Monte Carlo settings
☒	☐	For hierarchical and complex designs, identification of the appropriate level for tests and full reporting of outcomes
☐	☒	Estimates of effect sizes (e.g. Cohen's *d*, Pearson's *r*), indicating how they were calculated

Our web collection on statistics for biologists contains articles on many of the points above.

Software and code

Policy information about availability of computer code

Data collection	Microscopy pictures were acquired with Zeiss LSM 700 Upright or ZEISS LSM 980 with Airyscan 2 confocal microscope (Carl Zeiss AG); Victor X4 plate reader (Perkin Elmer) was used for all the assays requiring absorbance, luminescence or fluorescence quantifications; the qPCR reactions were performed using the Light-Cycler system (Roche Applied Science).<br><br>RNA-seq analysis was performed using the R (version 3.6.3), FastQC (version 0.11.9) was used to verify the quality of the sequence data. Sequenced reads were mapped using STAR aligner (version 2.6.0a). Reads were counted using htseq-count (version 0.10.0). Differential expression of genes was calculated by using Limma-Voom method.
Data analysis	GraphPad Prism 8 for Mac OS X (GraphPad Software, Inc.; v8.3.1), JMP (https://www.jmp.com/en_ch/home.html; v18.0.1), Fiji (http://imagej.nih.gov/ij; version 1.47b). Heat-maps were generated by using Morpheus (https://software.broadinstitute.org/morpheus). Motif enrichment analysis was performed with HOMER (v4.11). Functional clustering in this study was performed by using the DAVID (Database for Annotation, Visualization and Integrated Discovery) database (https://david.ncifcrf.gov/home.jsp; v6.8).

For manuscripts utilizing custom algorithms or software that are central to the research but not yet described in published literature, software must be made available to editors and reviewers. We strongly encourage code deposition in a community repository (e.g. GitHub). See the Nature Portfolio guidelines for submitting code & software for further information.

Data

Policy information about availability of data

All manuscripts must include a data availability statement. This statement should provide the following information, where applicable:

- Accession codes, unique identifiers, or web links for publicly available datasets
- A description of any restrictions on data availability
- For clinical datasets or third party data, please ensure that the statement adheres to our policy

Sequencing data that support the findings of this study have been deposited in the Gene Expression Omnibus under accession codes: GSE196021, GSE196022 and GSE296199. For all RNA-seq analyses, reads were mapped against the Caenorhabditis_elegans.WBcel235.89 genome downloaded from Ensembl. Gene expression data for the elt-2 OE worms were retrieved from the Gene Expression Omnibus (GSE69263). Uncropped images for immunoblots and statistical data are available as source data. All other relevant data and materials are available either in the article and Supplementary Tables or from the corresponding authors upon reasonable request.

Field-specific reporting

Please select the one below that is the best fit for your research. If you are not sure, read the appropriate sections before making your selection.

☒ Life sciences ☐ Behavioural & social sciences ☐ Ecological, evolutionary & environmental sciences

For a reference copy of the document with all sections, see nature.com/documents/nr-reporting-summary-flat.pdf

Life sciences study design

All studies must disclose on these points even when the disclosure is negative.

Sample size	No statistical methods were used to pre-determine sample sizes, but our sample sizes are similar to those reported in previous publications (Li, T. Y. et al., Nat Aging, 2021; Savini, M. et al., Nat Cell Biol, 2022; Houtkooper, R. H. et al. Nature, 2013).
Data exclusions	No data were excluded from the analysis, except for the C. elegans lifespan experiments (the reasons for censoring were the "exploded vulva" phenotype or worms that crawled off the plate). These reasons were pre-established before the beginning of the experiment (Li, T. Y. et al., Nat Aging, 2021; Savini, M. et al., Nat Cell Biol, 2022; Houtkooper, R. H. et al. Nature, 2013).
Replication	All experiments, except for the RNA-seq, were repeated at least twice and similar results were acquired.
Randomization	Except for the random allocation of C. elegans to experimental groups/treatments after large-scale synchronization, the experiments were not randomized.
Blinding	Investigators were not blinded to allocation during experiments and outcome assessment, except for the RNA-seq analyses (Figs. 1h, 3g and 5f), where data analysis was performed in a blind manner until the group-to-group comparison steps were reached.

Reporting for specific materials, systems and methods

We require information from authors about some types of materials, experimental systems and methods used in many studies. Here, indicate whether each material, system or method listed is relevant to your study. If you are not sure if a list item applies to your research, read the appropriate section before selecting a response.

Materials & experimental systems

n/a	Involved in the study	
☐	☒	Antibodies
☐	☒	Eukaryotic cell lines
☒	☐	Palaeontology and archaeology
☐	☒	Animals and other organisms
☒	☐	Human research participants
☒	☐	Clinical data
☒	☐	Dual use research of concern

Methods

n/a	Involved in the study	
☒	☐	ChIP-seq
☒	☐	Flow cytometry
☒	☐	MRI-based neuroimaging

Antibodies

Antibodies used	Western blots were carried out with antibodies against green fluorescent protein (GFP) (Cat. 2956, CST, 1:1000, RRID:AB_1196615), β-amyloid 1–16 (6E10) (Cat. 803001, BioLegend, 1:1000, RRID:AB_2564653), Tubulin (Cat. T5168, Sigma, 1:2000, RRID:AB_477579), H3K27Ac (Ab4729, abcam, 1:1000, RRID:AB_2118291), H3K9Ac (Cat. 06-942, 1:1000, Merck, RRID:AB_310308), H3K4Ac (Cat. Ab176799, abcam, 1:1000, RRID:AB_2891335), Histone 3 (Cat. 9715, CST, 1:2000, RRID:AB_331563). The antibody for worm CPL-1

(1:5000) was a kind gift from Prof. Xiaochen Wang (SUSTech), as described and validated previously (Sun, Y. et al. Elife, 2020). Horseradish peroxidase (HRP)-labeled anti-rabbit (Cat. 7074; CST, 1:5,000, RRID:AB_2099233) and anti-mouse (Cat. 7076; CST; 1:5,000, RRID:AB_330924) secondary antibodies were applied.

Validation

Anti-GFP (CST, Cat. 2956); Suitable for: WB, IHC; Reacts with: all species
https://www.cellsignal.com/products/primary-antibodies/gfp-d5-1-rabbit-mab/2956
Anti-β-amyloid 1–16 (6E10) (BioLegend, Cat. 803001); Suitable for: WB, ELISA, IHC-P; Reacts with: Human
https://www.biolegend.com/de-de/products/purified-anti-beta-amyloid-1-16-antibody-11228?GroupID=BLG15648
Anti-Tubulin (Sigma, Cat. T5168); Suitable for: WB, IF, RIA; Reacts with: Mouse, Chicken, Chlamydomonas, African green monkey, Human, Rat, Bovine, Sea urchin, Kangaroo rat
https://www.sigmaaldrich.cn/CN/zh/product/sigma/t5168
Anti-H3K27Ac (Abcam, Ab4729); Suitable for: ICC/IF, WB, IHC-P, ChIP, PepArr; Reacts with: Mouse, Rat, Cow, Human, Recombinant fragment
https://www.abcam.cn/products/primary-antibodies/histone-h3-acetyl-k27-antibody-chip-grade-ab4729.html
Anti-H3K9Ac (Merck, Cat. 06-942); Suitable for: WB, ChIP, DB, FC, ChIP-seq; Reacts with: Human, Mouse, Rat
https://www.merckmillipore.com/CN/zh/product/Anti-acetyl-Histone-H3-Lys9-Antibody,MM_NF-06-942
Anti-H3K4Ac (Abcam, Cat. Ab176799); Suitable for: WB, ICC/IF, DB, ChIP, PepArr, ChIP-seq; Reacts with: Mouse, Human
https://www.abcam.cn/products/primary-antibodies/histone-h3-acetyl-k4-antibody-epr16596-chip-grade-ab176799.html
Anti-Histone 3 (CST, Cat. 9715); Suitable for: WB; Reacts with: Human, Mouse, Rat, Monkey, Zebrafish, Bovine, Pig
https://www.cellsignal.com/products/primary-antibodies/histone-h3-antibody/9715
Anti-CPL-1; Suitable for: WB; Reacts with: C. elegans; Validated by the producer in their publication:
Sun, Y. et al. Lysosome activity is modulated by multiple longevity pathways and is important for lifespan extension in C. elegans. Elife 9:e55745, doi: 10.7554/eLife.55745 (2020).

Eukaryotic cell lines

Policy information about cell lines

Cell line source(s)	N.A.
Authentication	N.A.
Mycoplasma contamination	N.A.
Commonly misidentified lines (See ICLAC register)	N.A.

Animals and other organisms

Policy information about studies involving animals; ARRIVE guidelines recommended for reporting animal research

Laboratory animals

The N2 (Bristol) strain was employed as the wild-type strain. IA123 (ijIs10[cpr-5::GFP-NLS::lacZ + unc-76(+)]), CB1370 [daf-2(e1370)], CF1038 [daf-16(mu86)], VC222 [raga-1(ok386)], RB754 [aak-2(ok524)], DA465 [eat-2(ad465)], VC3201 [atfs-1(gk3094)], OP56 (gaEx290 [elt-2::TY1::EGFP::3xFLAG(92C12) + unc-119(+)]), CL2122 (dvIs15 [pPD30.38] unc-54(vector) + (pCL26) mtl-2::GFP]), GMC101 (dvIs100 [unc-54p::A-beta-1-42::unc-54 3′-UTR + mtl-2p::GFP]), AM140 (rmIs132 [unc-54p::Q35::YFP]), AM725 (rmIs290 [unc-54p::Hsa-sod-1(127X)::YFP]), DA2123 (adIs2122 [lgg-1p::GFP::lgg-1 + rol-6(su1006)]), GRU101(gnaIs1[myo-2p::yfp]), CA1200 (ieSi57 [eft-3p::TIR1::mRuby::unc-54 3'UTR + Cbr-unc-119(+)] II), JIN1375 [hlh-30(tm1978) IV], atfs-1(tm4525) V, SM190 [pha-4(zu225);smg-1(cc546ts)], GRU102 (gnaIs1[myo-2p::yfp + unc-119p::Aß1-42]), HZ1683 [atg-2(bp576)], HZ1684 [atg-3(bp412)], HZ1687 [atg-9(bp564)] and HZ1688 [atg-13(bp414)] were provided by the Caenorhabditis Genetics Center (CGC, University of Minnesota) or the National Bioresource Project (NBRP). The XW19180 [hsp-16.2p::nuc-1::pHTomato] strain was a kind gift from Prof. Xiaochen Wang (SUSTech).The MQD2491[daf-16(hq389[daf-16::gfp::degron]) I; ieSi57[eft-3p::TIR1::mRuby::unc-54 3′UTR+Cbr-unc-119(+)] II; unc-119(ed3) III; daf-2(e1370ts) III] strain was a kind gift from Prof. Meng-Qiu Dong (NIBS). Hermaphrodite and L4/young adult worms were used for analyses throughout the study, unless otherwise specified in the figure legends or methods.

For generation of the strains with GFP-tag of vha-1, vha-14, vha-15, vha-16 and vha-20, the constructs (vha-1, Clone: 9473457628999774 E12; vha-14, Clone: 8859124759762056 C08; vha-15, Clone: 3304493055384826 B08; vha-16, 2491680425634929 G12; vha-20, Clone: 5745981749165295 F12) were obtained from Prof. Mihail Sarov, as part of the TransgeneOme project (https://transgeneome.mpi-cbg.de/). The constructs were injected at 10-60 ng/μL along with a co-injection marker pRF4 (rol-6) at 40 ng/μL to generate transgenic lines. Strains were made by the SunyBiotech Co.

A new UV-integrated N2 background cpr-5 reporter strain TYL001 (cpr-5p::gfp + rol-6) for optimal LySR activation detection was also constructed, which is available upon request. To construct this strain, the 1018 bp cpr-5 promoter was amplified with the following primers: 5′- GAATTGACATGCACTCCGGC-3′ and 5′-AAGAATAGCGGAGAGCTTCC-3′ and ligated in frame with eGFP in a pPD95.75 expression vector (Addgene, #184130). The construct was then injected at 50 ng/μL along with a co-injection marker pRF4 (rol-6) at 40 ng/μL. Extra-chromosomal arrays were integrated using UV irradiation and backcrossed two times to N2, non-roller worms were maintained afterwards.

For generation of the TYL002 (ges-1p::cpr-5-DsRed::SL2::GFP + rol-6) worm strain, the ges-1 promoter (amplified from the pJL3 plasmid, Addgene #184131), the cpr-5 protein coding sequence (amplified from worm total cDNA) and DsRed sequence (amplified from the pJL6 plasmid, Addgene #184134), were ligated into a pPD95.77_SL2 vector backbone (Addgene #184129), between SphI and XmaI restriction sites. The construct was then injected at 25 ng/μL along with a co-injection marker pRF4 (rol-6) at 40 ng/μL to generate transgenic lines.

For generation of the knock-in worm strains with endogenously GFP/Degron-mNG tagged VHA-6 [TYL003 (vha-6p::vha-6::gfp) and

TYL004 (vha-6::Degron::mNG)] and ELT-2 [TYL005 (elt-2::Degron::mNG)], the CRISPR/Cas9 engineering was performed by microinjection using the homologous recombination approach (Paix, A. et al. Genetics, 2015). The microinjection mixture consisted of 300 mM of KCl, 20 mM of HEPES, 100 ng/µL of tracrRNA (Cat. U-002005, Dharmacon), 50 ng/µL of crRNA targeting vha-6 or elt-2, 200 ng/µL of DNA repair template for vha-6 or elt-2, 0.25 µg/µL of Cas9 protein (Cat. CAS9PROT-250UG, Sigma), 200 ng/µL dpy-10 crRNA and 200 ng/µL dpy-10 repair template. To generate the homologous recombination DNA repair templates, two homologous arms (~1000 bp each) corresponding to the 5'- and 3'- sides of the insertion site and the GFP/Degron-mNG tags were cloned in a vector and then amplified altogether. Plasmids were injected into the gonad of young adult hermaphrodite worms using standard method. F1s with roller phenotype were singled on a new NGM plate and allowed to produce sufficient offspring. Successful knock-in events were screened by PCR genotyping from independent F1 transgenic animals' progeny that did not display roller phenotype, and further confirmed by DNA sequencing. The crRNAs and cloning primers used to generate the two strains are listed in Supplementary Table 6.
For generation of the strains expressing vha-6 and elt-2 promoter-driven mCherry TYL006 (vha-6p::mCherry; vha-6p::vha-6::gfp) and TYL007 (elt-2p::mCherry; elt-2::Degron::mNG)], promoters of vha-6 or elt-2 were amplified with the following primers: 5'-TCGGTAAGTTGCTACTTCAG-3' and 5'-TTTTTATGGGTTTTGGTAGGTTTTAG-3' for vha-6 promoter; 5'-ATTATATGAAAACTAATGAG-3' and 5'-TCTATAATCTATTTTCTAGTTTCTATTTTATT-3' for elt-2 promoter. The PCR products were then ligated in frame with mCherry in a pPD95.75 expression vector (Addgene, #184130). The constructs were then injected into the gonad of their corresponding TYL003 (vha-6p::vha-6::gfp) and TYL004 (elt-2::Degron::mNG) strains at 50 ng/µL along with a co-injection marker pRF4 (rol-6) at 40 ng/µL.

Wild animals

No wild animals were used in the study.

Field-collected samples

No field collected samples were used in the study.

Ethics oversight

This study did not require an ethical approval.

Note that full information on the approval of the study protocol must also be provided in the manuscript.
