# Supplementary figures and images for: A lysosomal surveillance response to stress extends healthspan
Source: Nat Cell Biol. 2025 Jun 26;27(7):1083–97. doi: 10.1038/s41556-025-01693-y (PMC12270918; doi:10.1038/s41556-025-01693-y)

**Source Data Fig. 3. Uncropped western blots with size marker indications.**

Fig. 3d

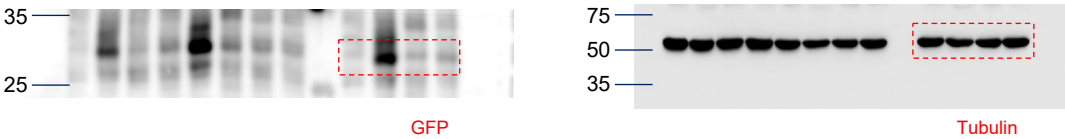

Fig. 3j

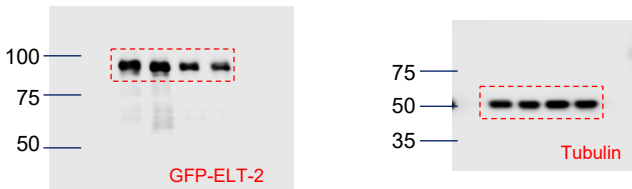

Supplement: Supplementary file 9 — Unprocessed western blots. [file 41556_2025_1693_MOESM9_ESM.pdf]

Source Data Fig. 4. Uncropped western blots with size marker indications.

Fig. 4e

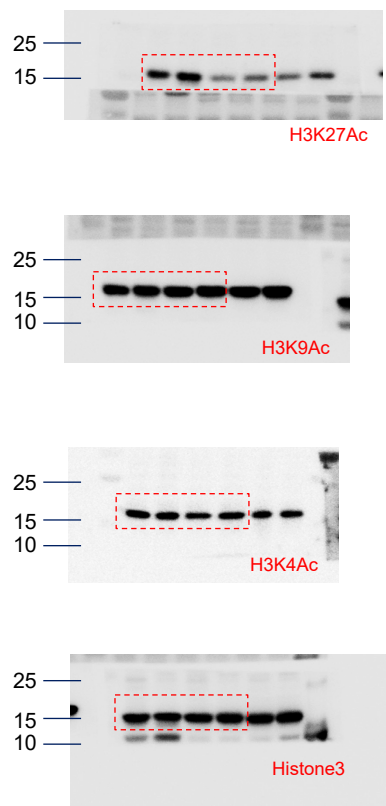

Supplement: Supplementary file 10 — Unprocessed western blots. [file 41556_2025_1693_MOESM10_ESM.pdf]

Source Data Fig. 6. Uncropped western blots with size marker indications.

Fig. 6b

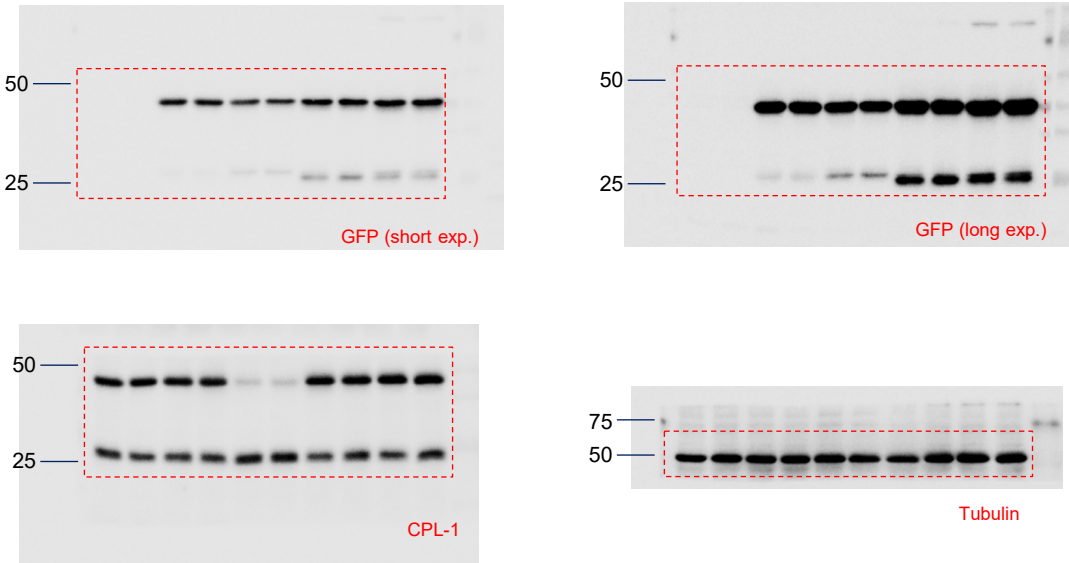

Supplement: Supplementary file 11 — Unprocessed western blots. [file 41556_2025_1693_MOESM11_ESM.pdf]

Source Data Fig. 7. Uncropped western blots with size marker indications.

Fig. 7b

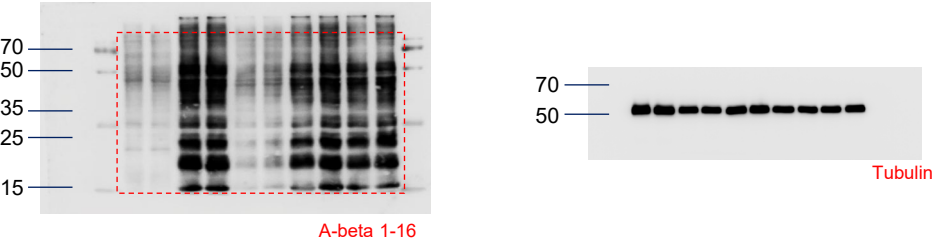

Fig. 7g

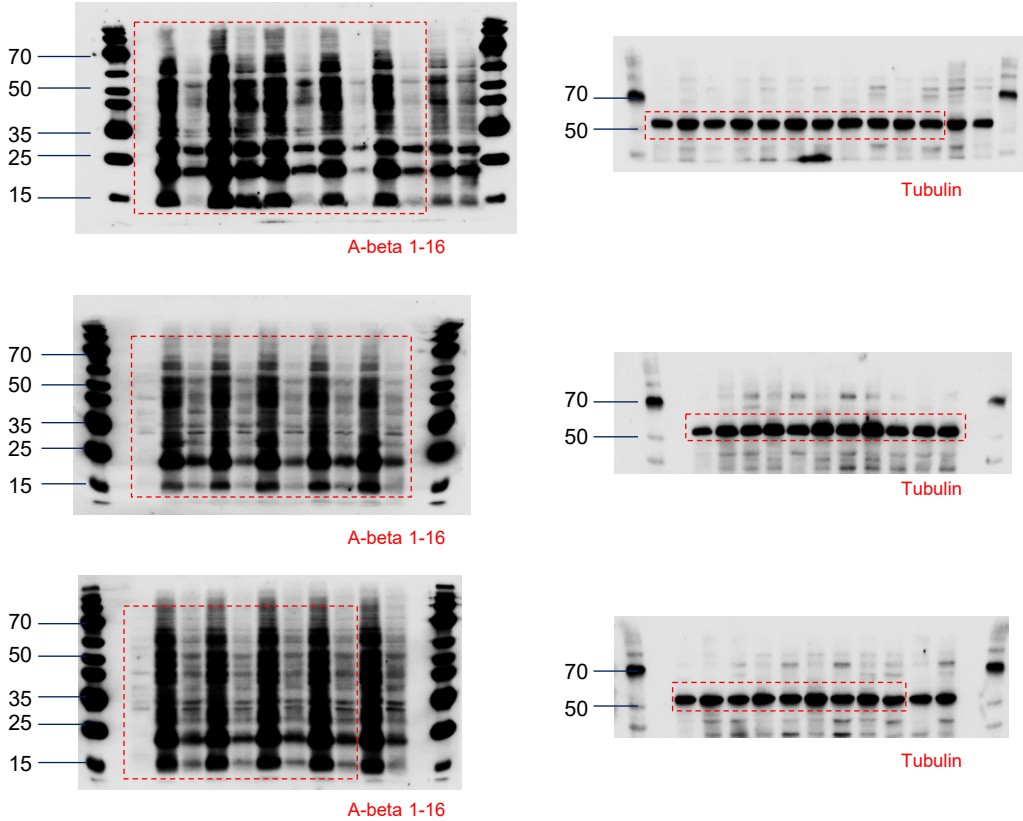

Supplement: Supplementary file 12 — Unprocessed western blots. [file 41556_2025_1693_MOESM12_ESM.pdf]
