# Supplementary material for: A lysosomal surveillance response to stress extends healthspan
Source: Nat Cell Biol. 2025 Jun 26;27(7):1083–97. doi: 10.1038/s41556-025-01693-y (PMC12270918; doi:10.1038/s41556-025-01693-y)

Source Extended Data Fig. 8. Uncropped western blots with size marker indications.

Extended Data Fig. 8b

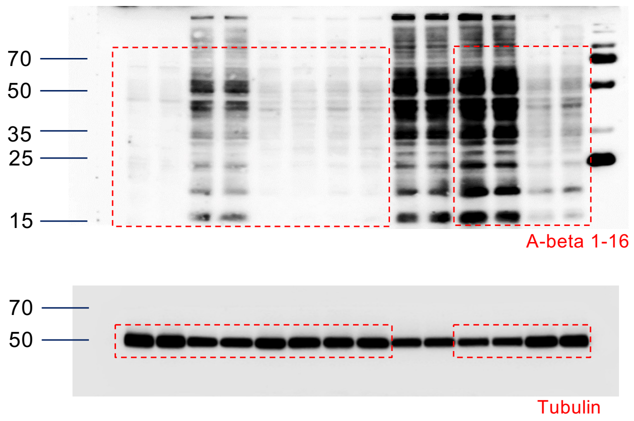

Extended Data Fig. 8c

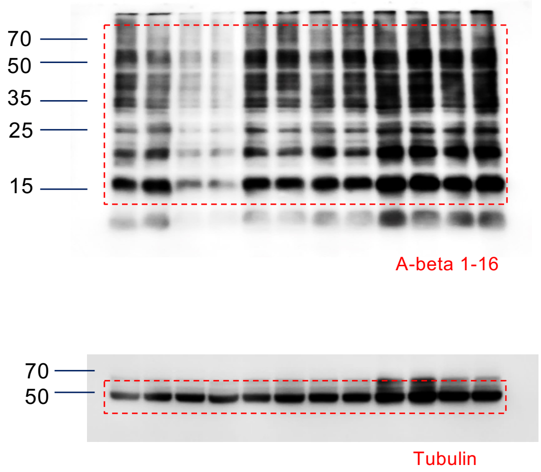

Supplement: Supplementary file 13 — Unprocessed western blots. [file 41556_2025_1693_MOESM13_ESM.pdf]
